# Effect of preoperative topical dexpanthenol moisturizer on the prevention of angular cheilitis after pediatric adenotonsillectomy: a prospective randomized controlled trial

**DOI:** 10.1007/s00405-025-09981-x

**Published:** 2026-01-21

**Authors:** Ethem İlhan, Fırat Onur, Bekir Salim Demir

**Affiliations:** Department of Otorhinolaryngology, University of Health Sciences, Gaziosmanpaşa Training and Research Hospital, Abdi İpekçi Street, 34255 Gaziosmanpaşa, İstanbul, Türkiye

**Keywords:** Adenotonsillectomy, Angular cheilitis, Dexpanthenol, Pediatrics, Pain, Moisturizer

## Abstract

**Purpose:**

To assess whether applying topical dexpanthenol to the oral commissures immediately before surgery reduces the incidence of postoperative angular cheilitis in children undergoing adenotonsillectomy.

**Methods:**

A total of 101 pediatric patients who underwent adenotonsillectomy were included in this study. Patients were randomly divided into two groups based on whether or not dexpanthenol was applied to the lip corners immediately before surgery. Postoperative pain and presence of angular cheilitis were assessed and compared between the groups on the postoperative first day and first week.

**Results:**

In the study group, only 9.8% (*n* = 5) of patients exhibited angular cheilitis on postoperative first day, whereas in the control group angular cheilitis incidence was 44% (*n* = 22) and the difference between two groups was statistically significant (*p* < 0.001). On postoperative first week examination, 3.9% (*n* = 2) of patients in the study group and 10% (*n* = 5) of patients in the control group exhibited angular cheilitis and the difference between the two groups was not significant (*p* > 0.05). Logistic regression analysis revealed that topical dexpanthenol application significantly reduced the odds of developing angular cheilitis on the first postoperative day (Odds Ratio: 0.14; 95% CI: 0.05–0.41; *p* < 0.001). Pain scores recorded on postoperative first day and first week did not show significant difference between the two groups (*p* > 0.05).

**Conclusions:**

A single application of topical dexpanthenol immediately before surgery is a simple intervention and significantly reduces the early incidence of postoperative angular cheilitis without affecting postoperative pain scores in pediatric adenotonsillectomy patients.

**Trial registration:**

ClinicalTrials.gov Identifier: NCT07002632, registered on 25 May 2025 (retrospectively registered).

**Supplementary Information:**

The online version contains supplementary material available at 10.1007/s00405-025-09981-x.

## Introduction

Tonsillectomy is a frequently performed operation in the pediatric population with or without adenoidectomy [1]. When appropriately indicated, adenotonsillectomy can enhance quality of life and promote child development [2, 3]. Postoperative hemorrhage and pain are the major factors associated with tonsillectomy morbidity [4, 5]. Irritation of free nerve endings, mucosal injury, inflammation of the tonsillar fossa, and pharyngeal muscle spasm are the primary causes of post-tonsillectomy pain [4, 6, 7].

Cheilitis is described as the inflammation of the lips. It may occur as an isolated condition or as a part of some systemic diseases [8]. A particular subtype, angular cheilitis (AC) which is also called angular stomatitis, typically manifests itself at the vermillion commissures of the mouth and adjacent mucous membranes [9]. Although AC can occur following tonsillectomy and adenotonsillectomy in clinical practice, the existing literature specifically addressing this postoperative complication is notably limited. Our clinical experience and anecdotal reports from colleagues suggest a considerable occurrence of AC after tonsillectomy, an observation that remains insufficiently explored in the literature. To our knowledge, there is only one study in the literature that addresses AC following tonsillectomy [4]. Mechanical trauma and dryness are probably the fundamental mechanisms contributing to the development of post-tonsillectomy AC and may be associated with pain and delays in return to normal diet, hence exacerbating morbidity [4]. Therefore, prevention of this condition after tonsillectomy may be important especially in pediatric patients.

Dexpanthenol is a derivative of pantothenic acid (vitamin B5) and widely used in dermatology for its regenerative and wound-healing properties [10]. The topical application of dexpanthenol stimulates skin regeneration and facilitates wound healing [11]. Dexpanthenol is a hygroscopic compound with significant water-binding ability, enhancing the hydration of the skin epithelium [12]. Therefore, topical dexpanthenol is employed to moisturize the skin, aiding in the preservation of skin softness and elasticity. It also enhances reepithelialization and exhibits anti-inflammatory effects [10].

The aim of this study was to ascertain whether topical dexpanthenol used during surgery can prevent the development of post-adenotonsillectomy AC. To our knowledge, this study is the first study that systematically evaluates the prophylactic topical intraoperative application of dexpanthenol for preventing post-adenotonsillectomy AC in children.

## Materials and methods

This prospective, single-center, assessor-blinded randomized, controlled study was designed and conducted in strict adherence to the ethical principles of the Declaration of Helsinki. The study protocol was approved by the institutional ethics committee and written informed consent was obtained from patients’ parents. However, the trial was retrospectively registered at ClinicalTrials.gov (Identifier: NCT07002632) on 25 May 2025, after completion of patient enrollment and prior to the formal statistical analysis because, at the time of study initiation, authors were not yet aware of the requirement for prospective registration of this type of interventional study. Crucially, the study protocol remained unchanged at any point between initiation of patient enrollment and trial registration.

This study was designed and reported in compliance with the CONSORT Statement guidelines for randomized controlled trials, with particular attention to allocation concealment and assessor blinding. A CONSORT flow diagram illustrating the progress of participants through the trial phases is included. A formal power analysis was not performed since there was no reliable pilot data on the presence of AC associated with moisturizer use in patients after tonsillectomy/adenotonsillectomy. The only study in the literature by England et al. [4] demonstrated non-significant results for the moisturizing variable, making the calculation of a reliable effect size impossible. Consequently, we initiated the study with a pragmatic sample size of at least 60 participants. However, patient enrollment was carried on after the recruitment of 60 patients throughout the study period to optimize statistical power and improve the reliability of the findings.

A total of 106 children aged 3–15 years scheduled for adenotonsillectomy between July 2021 and July 2024 at a tertiary referral ENT facility were enrolled in the study. Patients were randomized into study and control groups using a computer-generated random number list with a 1:1 allocation ratio. In the study group, a water-in-oil emulsion formula containing 5% dexpanthenol (Bepanthol^®^ Derma moisturizing cream) was applied to both oral commissures as a moisturizer immediately before mouth gag insertion while the control group received no cream. The Brodsky Grading Scale was used to assess preoperative tonsillar hypertrophy. Preoperative Mallampati scores of patients were also recorded. The conventional cold steel tonsillectomy technique was preferred in all patients to ensure consistency. Under the supervision of the same senior surgeon, all surgeries were performed by the same resident. Under general anesthesia, the Boyle-Davis mouth gag was inserted. To ensure standardization, Boyle-Davis mouth gag frame (Aesculap, Inc., Tuttlingen, Germany) was used for all surgical procedures. Only the size of the tongue depressor blade (Aesculap, Inc., Tuttlingen, Germany) attached to the gag was individualized according to mouth size and meticulously recorded for each patient. Care was taken to avoid gross lip entrapment between the gag and the dentition, and no additional standardized lip-protection devices or ointments were used apart from the study intervention in the dexpanthenol group. Prior to incision, the distance between the upper and lower central incisors was measured using a ruler. Adenoidectomy was performed first using a standard curette technique under direct visualization with a mirror. Following excision of adenoid tissue, gauze packs were placed in the nasopharynx to achieve hemostasis and bipolar cautery was applied when necessary. Subsequently, tonsillectomy was performed using the cold steel dissection technique. Once the pericapsular plane was identified, the tonsils were dissected bluntly and excised. Bipolar electrocoagulation and nonabsorbable 2.0 silk ties were utilized simultaneously to manage hemorrhage in all surgeries. The inferior tonsil poles were consistently ligated with 2.0 silk ties. The surgical time was recorded. A single dose of prophylactic cefazolin sodium (25 mg/kg) was administered parenterally to all patients preoperatively, and in the postoperative period, all patients received oral paracetamol suspension (20 mg/kg) three times daily for pain control.

Postoperative outcome assessments were performed by another senior surgeon blinded to group assignment. On postoperative first and seventh days, patients were assessed for the presence of AC and the severity of pain. The presence of AC was assessed clinically by inspection of the lip commissures and recorded as a binary outcome (present or absent); no formal AC severity or lip injury scoring scale was applied. Pain severity was assessed by using the Wong–Baker Faces Pain Rating Scale which ranges from 0 to 10 points. This scale reflects the patients’ overall postoperative pain, mainly related to oropharyngeal surgical sites. However, the scale did not allow for the specific isolation or scoring of pain originating from AC lesions at the lip commissures.

### Statistical analysis

Statistical analyses were performed in Statistical Package for the Social Sciences (SPSS) version 26.0 (IBM Corp., Armonk, NY, USA). Categorical variables were presented as frequencies and percentages (%). Numerical variables were expressed as mean and standard deviation (SD). The comparison of categorical variables was performed using the Pearson Chi-Square test. The normality of numerical variables was assessed using the Kolmogorov-Smirnov test, and variables following a normal distribution were compared using the Independent Samples T-Test. Binary logistic regression analysis was used to evaluate the independent effect of topical dexpanthenol use on the occurrence of AC on postoperative first day and first week. Odds ratios (ORs) with 95% confidence intervals (CIs) were calculated. The model’s explanatory power was assessed using Nagelkerke R². A p-value less than 0.05 was considered statistically significant.

## Results

A total of 106 pediatric patients who underwent adenotonsillectomy were included in this study. However, five patients did not adhere to the follow-ups and thus were excluded from the study. The study group included 51 patients, whereas the control group included 50 patients. The characteristics of patients in the study and control groups are presented in Table 1. There was no significant difference between study and control groups in terms of age, gender, tonsil grade or surgical indication (*p* > 0.05). The Kolmogorov-Smirnov test showed that the data were normally distributed.

**Table 1:** Characteristics of patients in study and control group

Characteristics		Control group (Dexpanthenol not used) (*n*:50)		Study group (Dexpanthenol used) (*n*:51)		*p*
		*n*	%	*n*	%	
Gender	Female	20	40.0	25	49.0	0.477
	Male	30	60.0	26	51.0	
Indication	Obstructive sleep apnea (OSAS)	13	26.0	14	27.5	0.521
	Recurrent adenotonsillitis	6	12.0	10	19.6	
	Recurrent tonsillitis + OSAS	31	62.0	27	52.9	
Tonsiller grade	Grade 1	7	14.0	8	15.7	0.952
	Grade 2	14	28.0	14	27.5	
	Grade 3	16	32.0	18	35.3	
	Grade 4	13	26.0	11	21.6	
		Mean ± S.D		Mean ± S.D		
Age^t^		5.96 ± 2.82		6.65 ± 2.36		0.187

*S.D* Standard Deviation. *Dexpant* Dexpanthenol, *t* Independent Sample T test. A *p*-value < 0.05 was considered statistically significant.

The comparison of the surgical parameters assessed are presented in Table 2. No significant difference was found between the study and control groups concerning these variables (*p*** > **0.05).

**Table 2:** Comparison of surgical parameters between study and control groups

Surgical parameters		Control group (Dexpanthenol not used) (*n*:50)		Study group (Dexpanthenol used) (*n*:51)		*p*
		*n*	%	*n*	%	
Mallampati classification	class 1	39	78.0	43	84.3	0.577
	class 2 class 3 class 4	11 0 0	22.0 0 0	8 0 0	15.7 0 0	
		Mean ± S.D		Mean ± S.D		
Vertical mouth opening length^t^		4.63 ± 0.89		4.48 ± 0.70		0.360
Mouth opener size^t^		7.01 ± 1.15		7.36 ± 0.76		0.071
Operation time (minutes)^t^		38.86 ± 18.53		35.10 ± 14.39		0.258

*S.D* Standard Deviation. *Dexpant* Dexpanthenol, *t* Independent Sample T test. A *p*-value < 0.05 was considered statistically significant

**Fig. 1:**
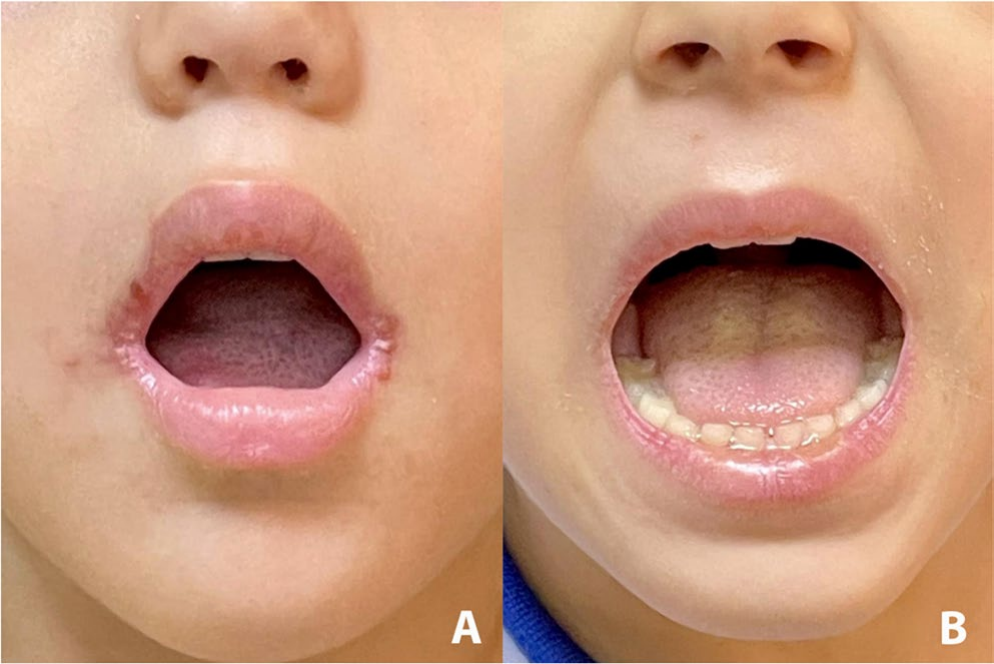
(**a**) A patient presenting with angular cheilitis, on postoperative first day following adenotonsillectomy. (**b**) Another patient, who received intraoperative topical dexpanthenol on lip commissures, showed no sign of angular cheilitis on postoperative first day following adenotonsillectomy

The severity of pain and the presence of AC in both study and control groups were assessed on the first postoperative day and the first week, and are presented in Table 3. On the first postoperative day only 9.8% (*n* = 5) of patients in the study group experienced AC compared to 44% (*n* = 22) in the control group, and the difference between the two groups was statistically significant (*p* < 0.001) (Fig. 1). Besides, AC was present in 3.9% (*n* = 2) of the study group and 10% (*n* = 5) of the control group on the postoperative first week assessment and the difference between the groups was not statistically significant (*p*** > **0.05). When the postoperative first day and first week pain scores in study and control groups were compared, there was no significant difference (*p*** > **0.05).

**Table 3:** Pain severity and existence of angular cheilitis in study and control groups

Angular cheilitis and pain presence		Control group (Dexpanthenol not used) (*n*:50)		Study group (Dexpanthenol used) (*n*:51)		*p*
		*n*	%	*n*	%	
1st day angular cheilitis	No	28	56.0	46	90.2	< 0.001*
	Yes	22	44.0	5	9.8	
1st week angular cheilitis	No	45	90.0	49	96.1	0.269
	Yes	5	10.0	2	3.9	
		Mean ± S.D		Mean ± S.D		
1st day pain score^t^		4.48 ± 1.94		4.47 ± 1.93		0.981
1st week pain score^t^		2.38 ± 1.59		2.41 ± 1.66		0.873

*t* Independent Sample T test, *S.D* Standard Deviation, *Dexpant* Dexpanthenol. A *p*-value < 0.05 was considered statistically significant

Logistic regression analysis demonstrated that topical dexpanthenol application was associated with a significantly reduced likelihood of developing AC on the first postoperative day (odds ratio: 0.14; 95% CI: 0.05–0.41; *p* < 0.001), corresponding to an 86% reduction in odds compared to no moisturizer application. However, dexpanthenol use did not significantly affect the incidence of AC at postoperative first week (*p*** > **0.05) (Table 4).

**Table 4:** Evaluation of the effect of dexpanthenol use on angular cheilitis with logistic regression analysis

Dependent variable	Independent variable	B	S.E.	*p*	Exp(B)/ Odds ratio	Confidence intervals 95 C.I.for EXP(B)		
						Min	Max	*R* ^2^
1st day angular cheilitis	Dexpanthenol used	-1.98	0.55	< 0.001*	0.14	0.05	0.41	0.213
1st week angular cheilitis	Dexpanthenol used	-1.00	0.86	0.245	0.37	0.07	1.99	0.037

*Exp(B)* Odds Ratio (OR), *B* Regression Coefficient, *S.E* Standard Error. A *p*-value < 0.05 was considered statistically significant

None of the patients in either the study or control group experienced any adverse events, such as allergic reactions or other complications, associated with the application of topical dexpanthenol or control procedure.

## Discussion

AC is a painful and inflammatory condition of the oral commissures that can cause significant patient discomfort [9, 13, 14]. Several local and systemic factors are proposed to contribute to the etiology of AC [8, 9, 14, 15]. Iatrogenic factors, including orthodontic treatment and tonsillectomy, have also been suggested as potential causes of AC [14, 15]. The tonsillectomy procedure requires adequate mouth opening during surgery, and surgeons use various mouth gag instruments to ensure appropriate mouth opening. Prolonged retraction, continuous friction and contact from surgical instruments at the oral commissures can lead to dryness and irritation of the lip corners. As a result, the development of AC may potentially occur [4].

In our tertiary research and educational hospital, tonsillectomy/adenotonsillectomy procedures are often performed by residents under supervision. Our clinical observations suggest that AC occurrence following tonsillectomy/adenotonsillectomy is not uncommon and sometimes is underestimated. Although commonly observed in surgical training practice, this issue has received almost no attention in the literature. In practice, preventive measures for this condition are also frequently overlooked. This study employed a 5% dexpanthenol cream to the lip commissures and assessed its preventive effect on the occurrence of AC following adenotonsillectomy. Dexpanthenol’s known moisturizing, barrier-restoring, and anti-inflammatory properties make it a suitable candidate for AC prevention [16–19].

To our knowledge, this is the first study to evaluate the role of intraoperative topical use of dexpanthenol for prevention of AC following adenotonsillectomy. A single intraoperative application of topical dexpanthenol to the oral commissures significantly reduced AC incidence on postoperative first day compared to the control group. This preventative effect seems to be related with improved hydration and epithelial barrier support at the lip corners associated with dexpanthenol use during surgery. Although a preventive effect was observed, the occurrence of AC in a small portion of patients in the study group suggests that unmeasured individual factors such as skin type or sensitivity might also have contributed to its development.

Although literature lacks data about the incidence of AC following adenotonsillectomy, the overall incidence of AC in this study may be high compared to general clinical experience. This observation may be partially attributed to our institution’s status as a tertiary training hospital, where procedures were performed by residents under senior supervision. Subtle variations in surgical technique or prolonged retraction during surgery might have contributed to local tissue trauma. Furthermore, the routine use of bipolar cautery and ligation for hemostasis, might have increased the risk of mechanical trauma to the lip commissures.

Additionally, the mean surgical duration in our study was longer than that reported in prior studies [20–22], which may theoretically increase exposure and drying of the lip corners. Several factors likely account for the longer surgical durations observed in our cohort. First of all, adenoidectomy was performed in all patients along with tonsillectomy. Also, the conventional cold steel tonsillectomy technique was preferred, with bipolar diathermy employed solely for hemostatic control in conjunction with ties, in accordance with the clinic’s bleeding control protocol. The longer surgical time may be attributed to our use of the cold steel technique, which is often slower than hot techniques [23]. As mentioned earlier, procedures were carried out by a resident under close supervision, which might have prolonged operation time due to surgical training. However, in our cohort surgical time did not differ significantly between study and control groups and was not shown to independently affect AC occurrence. However, prolonged mouth opening as a possible risk factor for AC should be evaluated more rigorously in future studies.

According to a study by England et al., it was reported that diathermy use was the only variable significantly associated with AC occurrence and use of diathermy increased the likelihood of developing AC 3.5 times. Moreover, the probability of developing AC seemed to increase with operation difficulty level [4]. Furthermore, they reported that AC caused significant discomfort in 30% of the subjects which was assessed with direct questioning to patients. It should be noted the mean age of patients in their study was 20.65 years, ranging from 7 to 56 years, which allowed the authors to specifically assess the AC associated discomfort. Although no specific information was provided about how and which moisturizer was used, there was no significant difference in terms of AC development with and without moisturizer use [4]. These findings must be interpreted cautiously, given the study’s small sample size and absence of randomization. In contrast, our randomized prospective study included a larger sample size, and, distinct from their results, we found no significant difference in terms of pain severity between the study and control groups. Due to the pediatric population enrolled in this trial, postoperative pain was assessed using the Wong–Baker Faces Pain Rating Scale as a global measure of overall oropharyngeal pain rather than a lip specific pain. It is possible that intense pharyngeal pain in the early postoperative period obscured milder lip discomfort associated with AC, especially in younger children who may struggle with pain localization. Moreover, the use of a subjective generic pain scale and the binary assessment of AC might have limited our ability to detect subtle differences in lip related symptoms. Conversely, AC may not have resulted in a significant measurable increase in the overall pain scores in every case, and this issue should be clarified in future studies using lip specific symptom assessments.

This study has some limitations. The sample size was determined without a formal power calculation due to the lack of literature which may limit the generality of our results. Future randomized trials with formal sample size estimation based on the observed incidence rates are warranted. As mentioned previously, AC related pain could not be determined specifically due to the generic pain scale used and the lack of lip specific pain assessments. Moreover, AC grading was not performed which also limited to identify variations in lip related symptoms. Furthermore, all patients were administered oral paracetamol suspension, which may have influenced individual pain severity perception and subsequently impacted our pain assessments. Finally, the trial was retrospectively registered at ClinicalTrials.gov, representing a methodological limitation.

## Conclusion

Intraoperative application of dexpanthenol to the lip commissures during adenotonsillectomy is a simple, safe, and cost-effective intervention that significantly reduces the incidence of AC in the immediate postoperative period. Our findings indicate that the topical intraoperative application of dexpanthenol cream is effective in preserving the integrity of the epithelial layer at the oral commissures during adenotonsillectomy, thereby preventing the development of AC. Further research is necessary to determine the factors associated with the occurrence of AC and the effectiveness of alternative moisturizing agents in reducing AC incidence following adenotonsillectomy.

## Supplementary Information

Below is the link to the electronic supplementary material.

Supplementary Material 1

## Data Availability

The data supporting the findings of this study are available within the article. Additional information can be obtained from the corresponding author upon reasonable request.

## Declarations

### Clinical trial registration

ClinicalTrials.gov Identifier: NCT07002632, registered on 25 May 2025 (retrospectively).
