# Supplementary material for: Effect of preoperative topical dexpanthenol moisturizer on the prevention of angular cheilitis after pediatric adenotonsillectomy: a prospective randomized controlled trial
Source: Eur Arch Otorhinolaryngol. 2026 Jan 21;283(4):2467–73. doi: 10.1007/s00405-025-09981-x (PMC13053382; doi:10.1007/s00405-025-09981-x)
Supplement: Supplementary file 1 — Supplementary Material 1 [file 405_2025_9981_MOESM1_ESM.pdf]

Figure 1: CONSORT 2025 Flow Diagram

Flow diagram of the progress through the phases of a randomised trial of two groups (that is, enrolment, intervention allocation, follow-up, and data analysis)

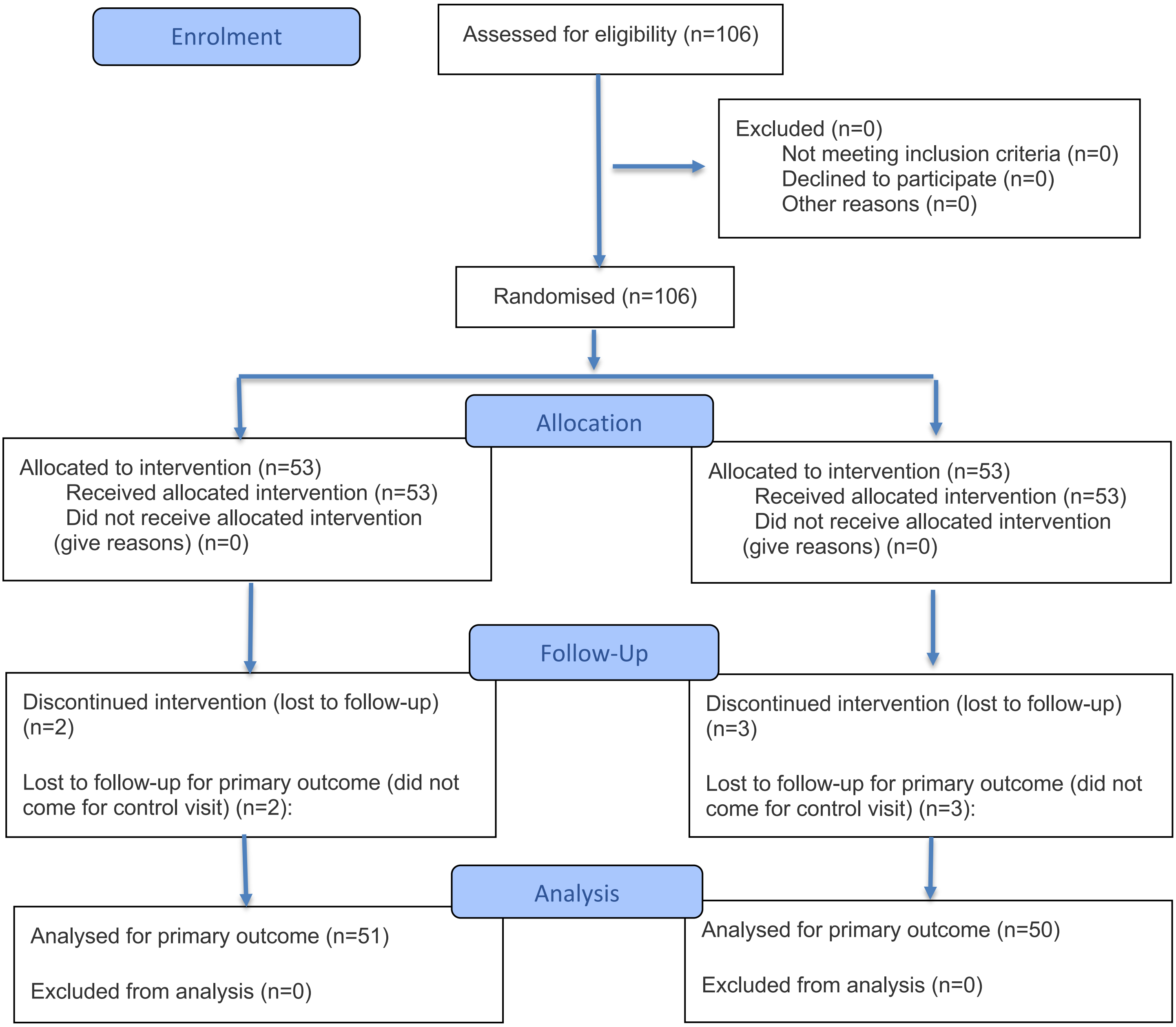

Citation: Hopewell S, Chan AW, Collins GS, Hróbjartsson A, Moher D, Schulz KF, et al. CONSORT 2025 Statement: updated guideline for reporting randomised trials. BMJ. 2025; 388:e081123. https://dx.doi.org/10.1136/bmj-2024-081123
© 2025 Hopewell et al. This is an Open Access article distributed under the terms of the Creative Commons Attribution License (https://creativecommons.org/licenses/by/4.0/), which permits unrestricted use, distribution, and reproduction in any medium, provided the original work is properly cited.
